# Prognostic Factors for Development of Subsequent Metastases in Localized Osteosarcoma: A Systematic Review and Identification of Literature Gaps

**DOI:** 10.1155/2020/7431549

**Published:** 2020-03-18

**Authors:** Patrick Basile, Emily Greengard, Brenda Weigel, Logan Spector

**Affiliations:** ^1^Department of Pediatric Hematology/Oncology, University of Minnesota, Minneapolis, MN, USA; ^2^Department of Epidemiology, University of Minnesota, Minneapolis, MN, USA

## Abstract

**Aim:**

To investigate prognostic factors in pediatric and young adult patients with localized osteosarcoma that could predict the development of subsequent pulmonary metastases and lead to an ability to risk-stratify therapy. We performed a systematic review of the literature published since January 1990 to establish common evidence-based prognostic factors.

**Methods:**

PubMed and Embase searches (Jan 1990–Aug 2018) were performed. Two reviewers independently selected papers for patients with localized osteosarcoma with subsequent metastatic development and then reviewed for quality of methods and prognostic factors.

**Results:**

Database searches yielded 216 unique results. After screening, 27 full-text articles were studied in depth, with 9 items fulfilling predetermined inclusion and exclusion criteria. Age, tumor location, tumor size/volume, and histologic response carried independent prognostic value in the majority of the studies.

**Conclusions:**

Several prognostic factors seemed to be consistent amongst the studies, but the heterogeneity and smaller sizes of the study populations made pooling of results difficult. Standardization of larger patient populations and consistent definitions/cutoffs for prognostic factors are needed to further assess for consistent prognostic factors and potential predictive models to be developed.

## 1. Introduction

High-grade osteosarcoma accounts for approximately 5% of all childhood malignancies with an incidence rate of 4.4 cases per million in patients aged 0–24 [1]. Historically, patients with localized disease treated with only surgical resection had poor outcomes with a 20% 3-year disease-free survival (DFS) [2]. Overall survival has increased since the 1970s from approximately 15%–70% with the addition of adjuvant and neoadjuvant chemotherapy to local control surgery [2]. One of the most important prognostic factors impacting survival rates is the presence of metastasis [3]. The most likely destination for metastatic disease is the lungs, although other locations such as bone are possible. Approximately 50–60% of those diagnosed with osteosarcoma develop metachronous metastases and 20% develop synchronous metastases [4]. Survival is influenced similarly if metastases are present at initial diagnosis or if they develop at subsequent follow-up evaluations [5].

Regardless of the timing for the development of metastatic disease, treatment for those with metastatic disease has been difficult. Although treatment regimens for synchronous lesions have been tolerable, they have not increased the survival or event-free survival rates (EFS) to nonmetastatic percentages [6]. Current standard of care for metastatic disease remains the same as localized disease with neoadjuvant chemotherapy, resection of the primary tumor, and adjuvant chemotherapy. Although 10% of pulmonary metastases will resolve after chemotherapy [7], the majority will require surgical resection following the completion of chemotherapy. This standard of care regimen has improved survival to approximately 70% [2], but also has been shown to have significant late effects such as cardiac insufficiency, hearing loss, and infertility [8].

Several different prognostic factors for overall survival (OS) when standard of care chemotherapy regimens containing platinum agents, doxorubicin, high-dose methotrexate, and ifosfamide, are used have been discussed in the literature [9–11]. Although risk factors for the development of subsequent metastases in patients have been evaluated, these have not been systematically reviewed.

The aim of our systematic review was to identify trends in the literature of the most commonly evaluated risk factors for metachronous development of metastases in those patients with localized disease at diagnosis. Early identification of these patients could lead to improvement in risk stratification and judicious use of chemotherapy.

## 2. Methods

MEDLINE and Embase were searched for eligible studies published in English between January 1990 and August 2018 and the month before the search was run. We used the following search strategy: Osteosarcoma AND metastasis AND risk AND factors AND (Humans[Mesh] AND (infant[MeSH] OR child[MeSH] OR adolescent[MeSH])) and limited to human studies. Reports were included if patients were <41 years old with initial diagnosis of nonmetastatic high-grade osteosarcoma at diagnosis. Studies were excluded if they included patients ≥41 years old (regardless if age groups were reported separately) or if metastatic disease was present at diagnosis. Retrospective case control studies, randomized control trials, and retrospective cohort studies were included in the review.

Quality assessment was completed evaluating manuscripts based on the defined inclusion and exclusion criteria as well as clear cohorts in the studies that included patients that had local disease at diagnosis and developed subsequent metastases. The qualitative review for risk factors was completed by Basile and Greengard with a third reviewer assigned (Spector) to resolve discordant reviews. Studies fulfilling the specified criteria were then reviewed for associations for risk of development of subsequent pulmonary metastatic lesions.

## 3. Results

**Figure 1:**
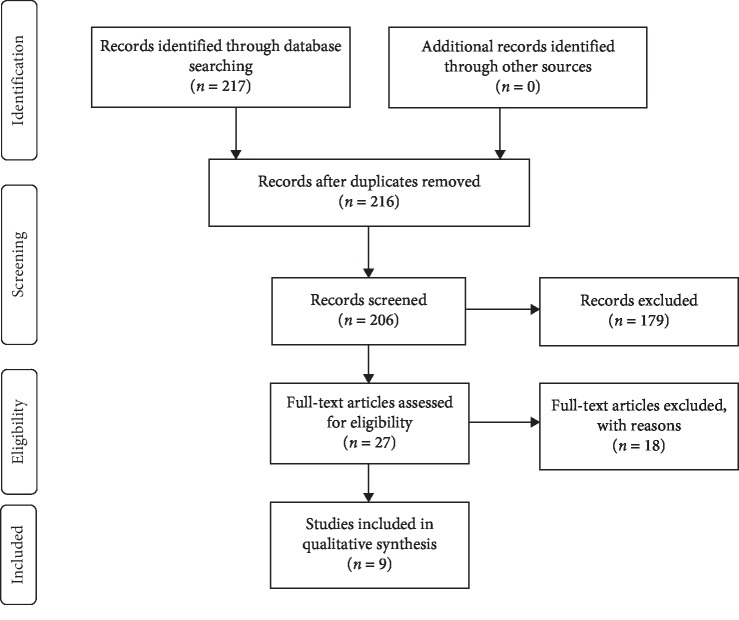
PRISMA flow diagram for the systematic review detailing the database searches, the number of abstracts screened, and the full texts retrieved.

The search resulted in a total of 217 publications and 216 after duplicates were removed. 206 records were screened after having abstracts available in English and 179 were excluded on abstract review. Of the 27 full text articles assessed for eligibility, 19 were excluded for not evaluating for subsequent metastases and not isolating analysis to patients with local disease, discussing multiple tumor types without isolation of osteosarcoma or not having full text available to review. This left 9 studies included in the qualitative synthesis (Figure 1). Duplicate authors were noted, but overlapping patient populations were not present upon detailed review.

### 3.1. Characteristics of Included Studies and Prognostic Factors

Most studies were retrospective in nature, and sample size varied from 19 to 2680 patients. One study [12] was a prospective study. This led to significant heterogeneity in sample sizes and also the majority of the sample sizes were small. All other articles' subjects were derived from a single institutional patient population or from international collaboration datasets [12, 13]. There was heterogeneity amongst the clinical prognostic factors that were evaluated for their association with subsequent metastasis, but several factors were evaluated in the majority of the studies: age, gender, tumor location, tumor size/volume, histologic subtype, and histological response (Table 1). Multivariate analysis (MVA) was performed in the reviewed papers when univariate significance was noted.

Among these common factors, there was substantial variation in definitions and cut off values used for the measurement of variables of interest. Age ranges varied greatly among studies. Tumor location definitions were often very broad or very specific; for example, Kong et al. [14] used humeral vs other, while Ward et al. [19] delineated areas by bone and proximal vs distal. Studies were varied on the use of either tumor size or tumor volume measurements, and the cutoffs used to assess risk varied study to study. Histology was evaluated as per pathological diagnosis in all instances, but like tumor location, the types were either somewhat broad (osteoblastic vs nonosteoblastic) or somewhat specific (osteoblastic vs chondroblastic vs telangiectatic etc.). Percent necrosis was relatively consistent using a single stratification point of 90%; however, some studies further stratified above 90% while others further substratified based on the Salzer-Kuntschick grading scale [21].

### 3.2. Age/Gender

Most of the studies evaluated age at diagnosis as a risk factor for the development of metachronous metastases. These studies used a variety of age cutoffs for their analysis. Kong et al. [14] and Bispo Júnior and Camargo [15] used a single cutoff of 15 years, while Smeland et al. [12] had a gender specific age cutoff of either 11 or 12 years to distinguish between children and adolescents in girls and boys, respectively. Hauben et al. [13] used 5-year increments to stratify the risk of metastatic recurrence. Association of age with risk of metastases development was not consistent among the studies; however, it was found to be a significant factor after MVA in EFS in the one prospective study that was included [12], demonstrating an improved survival for younger patients. Sex was included in 6 of the 9 studies, but did not show any prognostic significance with metastases development in univariate analysis (UVA).

### 3.3. Tumor Location/Tumor Size/Histological Subtype

Location of the primary tumor was also evaluated in the majority of studies (7/9), but had variance in its estimated impact on metachronous metastases development. Studies by Kim et al. [16] and Smeland et al. [12] showed that primary tumor location in the proximal humerus had an increased risk of metastases.

Tumor size/volume showed a slightly stronger association than tumor location. Three of the studies demonstrated an increased risk of metastases after MVA. However, there were differences in both the type of measurements and cutoffs used for the analyses. Two of the studies used volume; however, Kong et al. [14] used a hard cutoff of 150 mL while Smeland et al. [12] evaluated the relative cutoff of 1/3 the volume of the involved bone. Kim et al. [16] used maximal tumor diameter in their evaluation and nomogram development for the risk of metachronous metastases using evaluation of prognostic factors at diagnosis.

The histological subtype of these high-grade lesions did not appear to be significant in determining the risk of subsequent metastases. Seven of the studies performed a UVA with only two showing an impact. After MVA, only Bispo Júnior and Camargo [15] showed osteoblastic subtype to have an effect on metastasis-free survival (MFS) with worse rates of metastatic development compared to subtypes other than osteoblastic. This study did have a very low sample of 24 patients and only stratified the subtypes as osteoblastic and nonosteoblastic.

### 3.4. Surgical Margin/Histological Response

An uninvolved surgical margin on pathological review has historically been considered important to improving outcomes in osteosarcoma [13, 16, 22]. Four studies evaluated the association with Kong et al. [14] and Ward et al. [19] showing significance with both UVA and MVA. Both of these studies used a binary approach of involved/not-involved margins, while Bispo Júnior and Camargo [15] evaluated margins of 2 mm and Smeland et al. [12] used qualitative measures of “wide/radical, marginal, and intralesional.” The studies that showed significance agree with previous cooperative group studies that have shown that the width of the margin does not appear to be significant as long as the definitive biopsy/resection are performed at a center with orthopedic oncology experience [23, 24].

Six of the studies evaluated the prognostic factor of histological response to neoadjuvant chemotherapy, and all used a necrosis/cell death percentage of at least 90% as the cutoff for evaluation [12–14, 16, 19, 20]. Five of the studies found continued significance on MVA. Kong et al. [14] demonstrated a change on MFS on UVA while Hauben et al. [13] did not. This prognostic factor can be difficult to evaluate due to pathologist-to-pathologist variability in assessments. Also, given the heterogeneity of the studies and neoadjuvant therapy regimens varied (even in a single institutional study such as that by Ward et al. [19]) or were not clarified in the article [14]. Smeland et al. [12] performed a prospective study using the current standard of care of methotrexate, doxorubicin, and cisplatin which is accepted as the standard of care after increasing the EFS and overall survival to 54% and 71%, respectively.

### 3.5. Other Prognostic Factors

Several other factors were evaluated among the studies but were not included in more than 3 of the 9 studies. Pathologic fracture (3 studies), limb salvage vs amputation (1 study), time to seek care (1 study), elevated alkaline phosphatase (ALP) (1 study), and increased Bcl-2 expression and increased P-glycoprotein expression (1 study) did not show any significant changes in endpoint measurements in these individual studies.

## 4. Discussion

The goal of this review was to systematically evaluate the literature from 1990 to present to delineate the clinical/pathological risk factors that could be used to potentially predict which patients with localized osteosarcoma are more likely to develop subsequent metastases. The majority of studies were retrospective in nature, while some were designed to address prognostic factors directly and others did not have that direct intent. These studies had cohorts that were able to be reviewed, but not included in a meta-analysis mainly due to the heterogeneity in definitions/cutoffs of the different prognostic factors as well as treatment regimens being different or unclear. Also, while our initial intent was to concentrate on pediatric patients (i.e., <20 years of age at diagnosis), we subsequently opted to include studies that had patient populations less than 40 years of age due to rarity of the disease and to help increase the yield of the literature search. The studies were limited to primary osteosarcoma in order to reduce the risk of confounding by secondary disease. Histological response appeared to be the most consistent factor in prognosis for MFS in our study with suboptimal response to neoadjuvant therapy (<90% necrosis), carrying a significant increase in the risk of metastases development. The significance of this response is echoed in the recent Children's Oncology Group Study AOST0331 as the aim of the study was to intensify therapy in those patients with suboptimal response with the addition of ifosfamide and etoposide. However, therapy intensification did not improve outcomes and led only to increased toxicity for the subjects [12].

Other factors that demonstrated significance via MVA were age, tumor location, and tumor size/volume. Age is difficult to determine with any consistency among the studies, given the different cutoffs that were used between studies. Also, our inclusion of those studies with older patients could be skewing the data in those older age cutoff groups.

Tumor location was not found to be a significant factor in the majority of the studies it was evaluated in. Kim et al. [16] and Smeland et al. [12] showed significance for tumor location; however, Smeland et al. [12] combined both proximal humerus and proximal femur making it more difficult to delineate the effect. Interestingly, these were the same two studies that demonstrated significance with regards to age, raising the question of whether there is an association between age of presentation and site of primary lesion. The variation of definitions of location used in the studies may also have made this risk factor more difficult to evaluate. Some studies had very broad definitions with respect to location, while others were more specific to sections of certain bones. These variations may explain why poor prognostic factors, such as axial skeletal and pelvic primary lesions, may have been underappreciated in these studies [25].

Primary tumor burden not only had a varying definition in the form of measurement criteria (volume vs size) but also had varying cutoffs between studies that did use similar forms of measurement. As mentioned earlier, some used fixed measurements (150 ml) [14] while others used relative measurements (<1/3 of involved bone) [12]. The latter would be a more appropriate measure for pediatrics, given the differences in potential size of the patients; however, it would make data gathering potentially less consistent and more subjective.

Interestingly, two molecular/genetic factors were found on UVA to have significance but were only evaluated in two studies in our search. There are several other molecular/genetic factors that are found in the literature to potentially play a role in the outcome for patients with osteosarcoma, but consensus was clearly lacking. Based on our exclusion criteria, many of these studies were omitted as there was no data/analysis present on subsequent metastatic lesion development or patients had metastases at diagnosis. In the studies included in our review, Ferrari et al. [17] found that p53 mutations and absent ErbB-2 were associated with decreased recurrence free interval (RFI), but only in UVA and in a small sample size of 19 patients. Zhou et al. [18] were able to demonstrate that cytoplasmic HER-2 expression on osteosarcoma increased the risk of metastasis development. HER-2 is currently being evaluated as a therapeutic target for osteosarcoma in a phase 1 study using chimeric antigen receptor *T*-cells [26]. While there may be therapeutic potential with this receptor, its inclusion as prognostic factor for the development of metachronous metastases cannot be determined based on the current literature study.

Of the factors that were found to not have significance in the literature that was reviewed, presenting with a pathologic fracture at diagnosis was somewhat of a surprise. Pathologic fractures have been shown to potentially increase the risk of subsequent metastases while not affecting overall survival [27]. However, many articles were not included in our study as the metastatic status at diagnosis was not specified or there was no distinction between metachronous and synchronous metastases. This significantly limits our ability to truly assess the potential risk that pathological fracture may have on the development of metachronous metastases.

The development of a predictive nomogram by Kim et al. [16] was an encouraging exercise and took prognostic factors into consideration that were consistent in significance with our review. While the nomogram appeared to be accurate in predicting actual EFS, it was only bootstrapped with cohorts from the population that was used to develop the nomogram. Although the population was from a single institution, minimizing heterogeneity in several potential confounders also led to a smaller sample size being used as the basis for the nomogram. Kim et al. [28] is another Korean group that attempted to use a predictive nomogram and added in the use of ALP and capsular invasion. However, they had similar pitfalls with a small sample size of 141 patients and again consisted of a single ethnic group [28]. External validation of these nomograms with larger and minimally heterogeneous populations is required. The establishment of cooperative study groups such as the European and American Osteosarcoma Study (EURAMOS-1) will eventually lead to more standardized study populations and allow for these prognostic factors to be evaluated in a prospective manner, potentially serving as the basis for new nomograms or as a confirmatory resource for established nomograms.

The ability to predict the risk of metastatic disease in patients with localized osteosarcoma would be beneficial for several reasons. Most notably is a nomogram could be used for treatment stratification with the potential to spare low-risk patients the considerable toxicity associated with our current standard of care regimen. However, identification of these patients has been difficult, mainly due to issues with identifying consistent prognostic factors to develop a stratification nomogram similar to the one developed by Kim et al. [28] and Kim et al. [16]. During our literature search and review, several factors became apparent that emphasize potential issues when performing a systematic review or potential meta-analysis of a rare disease. First, the number of patients between the studies varies greatly and often times can be insufficient to draw significant and consistent conclusions regarding a particular prognostic factor. This was true for the majority of the literature included in our review, except for the Smeland et al. [12] study. Large collaborative studies such as EURAMOS-1 and those conducted by the Children's Oncology Group are absolutely necessary to obtain large pools of patients with rare diseases. While it is important to perform these prospective studies, it is critical to perform systematic reviews/meta-analyses to maximally use data from all available studies to draw more accurate conclusions and inform subsequent trial development.

The second major issue impacting the ability to combine analyses of published data is the discrepancy of measurement cutoffs and the definitions used for several of the prognostic factors. We recommend that future studies use consistent definitions of the following prognostic factors based on our literature review and experience:

1. Age should be used in sex-determined breaks due to typical differences in average ages of growth acceleration between the sexes. Smeland et al.'s [12] age cutoffs for children, adolescents, and adults were not too narrow as to cause smaller sample sizes but did distinguish differences in skeletal growth/maturity levels. Also, Jun et al. [29] did demonstrate evidence of increased growth velocity at diagnosis that was associated with worse outcomes.
2. Tumor location should have categories of femur/tibia, other limb bones to include humerus, and axial skeleton. Location of proximal/distal and metaphysis/diaphysis should also be included.
3. Tumor size should be determined by a consistent tumor length cutoff of <8 cm or ≥8 cm [16], with a relative length of the involved bone (greater than/less than 1/3 of the bone) also included.
4. The osteosarcoma subtype should be categorized by the histological subtypes of conventional chondroblastic, conventional osteoblastic, conventional other, telangiectatic, small cell, and high-grade surface osteosarcoma [12].
5. Surgical margin determination should be consistent using the Musculoskeletal Tumor Society (MSTS) originally defined by Enneking et al. [30], and this system classifies resection margins as intralesional (macroscopic or microscopic tumor at the margin), marginal (resection through the pseudocapsule or reactive zone around the tumor), wide (the presence of normal tissue between tumor/pseudocapsule and margin), or radical (entire anatomic compartment excised).
6. Histological response cutoff for adequacy of neoadjuvant chemotherapy should continue to be 90% tumor necrosis based on pathological determination. Central review of tissue samples should be incorporated in future clinical protocols to decrease as much interobserver variability from pathologist to pathologist as possible.

The development of metastases is a difficult obstacle to the successful treatment of osteosarcoma not only in persistence of disease but also in the need for more intensified therapies. Being able to predict which patients have an increased risk of subsequent metastases after upfront therapy, would potentially allow for therapy stratification after local control measures. Unfortunately, the literature is lacking in large-scale studies evaluating defined prognostic factors, limiting the creation of a predictive model of this rare disease difficult. The movement towards large-scale collaborative studies between organizations and countries will hopefully allow for the development of predictive models in the future, ultimately leading to more effective treatment.

## Figures and Tables

**Table 1:** Summary of the prognostic factors evaluated in the 9 studies included in the systematic review that included univariate or multivariate analysis.

	Kong et al. [14]		Bispo Júnior and Camargo [15]		Kim et al. [16]		Hauben et al. [13]		Ferrari et al. [17]		Zhou et al. [18]		Ward et al. [19]		Smeland et al. [12]		Ferrari et al. [20]	
	MFS (*N* = 114)		MFS (*N* = 24)		MFS (*N* = 365)		LRR (*N* = 33)		RFI (*N* = 19)		MFS (*N* = 19)		MFS (*N* = 111)		EFS (*N* = 1395)		DFS (*N* = 127)	
	UVA	MVA	UVA	MVA	UVA	MVA	UVA	MVA	UVA	MVA	UVA	MVA	UVA	MVA	UVA	MVA	UVA	MVA
Age^*∗*^	NS (15)	—	NS (15)	—	S (2; 15; 40)	S	NS (q5y)	—	—	—	—	—	—	—	S (sex dep)	S	NS (15; 21; 30)	—
Gender	NS	—	NS	—	NS	—	NS	—	—	—	—	—	—	—	NS	NS	NS	—
Tumor location	NS	—	NS	—	S	S	NS	—	—	—	—	—	NS	—	S	S	NS	—
Tumor size/volume#	S (v = 150 ml)	S	S (s = 15 cm)	NS	S (s = 6 cm; 8 cm)	S	—	—	—	—	—	—	—	—	S (v = 1/3 bone)	S (v)	NS	—
Osteosarcoma subtype	NS	—	S	S	NS	—	NS	—	—	—	—	—	NS	—	S	NS	NS	—
Surgical margin	S	S	NS	—	—	—	—	—	—	—	—	—	S	S	NS	NS	—	—
Histological response	S	NS	—	—	S	S	NS	—	—	—	—	—	S (90; 98)	S	S	S	S	S
HER-2/neu expression	—	—	—	—	—	—	—	—	—	—	S	—	—	—	—	—	—	—
P-glycoprotein	—	—	—	—	—	—	—	—	NS		—	—	—	—	—	—	—	—
ErbB-2	—	—	—	—	—	—	—	—	S	—	—	—	—	—	—	—	—	—
p53	—	—	—	—	—	—	—	—	S	—	—	—	—	—	—	—	—	—
BCL-2	—	—	—	—	—	—	—	—	—	—	—	—	—	—	—	—	—	—
Chemotherapy protocol	—	—	—	—	—	—	—	—	—	—	—	—	NS	S	—	—	S (MTX)	S (MTX)
Lactate dehydrogenase	—	—	—	—	—	—	—	—	—	—	—	—	—	—	—	—	S	S
Alkaline phosphatase	—	—	—	—	—	—	—	—	—	—	—	—	—	—	—	—	S	NS
Pathologic fracture	NS	—	—	—	NS	—	—	—	—	—	—	—	—	—	NS	NS		
Limb salvage vs amputation	—	—	NS	—	—	—	—	—	—	—	—	—	—	—	—	—		
Time to seek care	—	—	NS	—	—	—	—	—	—	—	—	—	—	—				

UVA = univariate analysis; — = not tested; MFS = metastasis-free survival; MVA = multivariate analysis; ^*∗*^ = cut off points in years in parenthesis; q5y = 5-year increments; ns = not significant (*P* < 0.05); EFS = event-free survival; # = measure in parenthesis (s = significant; (s) = size and (v) = volume).
